# The effects of a HEV‐filtering contact lens on the brightness of natural images

**DOI:** 10.1111/opo.70035

**Published:** 2025-10-29

**Authors:** Billy R. Hammond, John R. Buch, Patricia Martin, Wright Shamp, Jacob B. Harth, Cameron Wysocky, Lisa M. Renzi‐Hammond

**Affiliations:** ^1^ Vision Sciences Laboratory, Behavioral and Brain Sciences Program University of Georgia Athens Georgia USA; ^2^ Research and Development Johnson & Johnson Vision Care Inc. Jacksonville Florida USA; ^3^ Institute of Gerontology, College of Public Health University of Georgia Athens Georgia USA

**Keywords:** brightness, HEV filtering, natural images, randomised crossover trials, soft contact lenses, visual performance

## Abstract

**Purpose:**

Yellow filters, including high‐energy‐visible (HEV) filtering lenses, have been thought to increase the subjective brightness of real‐world scenes, but laboratory results are mixed. This study used a brightness matching technique modified to present natural images using a broad spectrum of wavelengths: a visibly clear control contact lens was compared to a contact lens containing a HEV‐filtering additive.

**Methods:**

A total of 121 subjects were tested (mean age = 33.8 ± 13.6 years). A stratified, controlled, prospective, randomised, double‐masked, bilateral, non‐dispensing, crossover design was used. Participants attended two study visits separated by a washout period and were randomly assigned to wear a HEV‐filtering contact lens or a clear control lens binocularly, counterbalanced on the second visit. Spherical (*n* = 41), multifocal (*n* = 40) and toric (*n* = 40) soft contact lenses were tested. Ten natural scenes were presented using a slide projector with a 550‐watt xenon light source. An adjustable comparison (broadband but short‐wave deficient), produced using parallel optics with a 1000‐watt xenon source, was varied based on the subject's judgement to match the brightness of the adjacent natural image. Three ascending/descending trials for each image were used to obtain the match.

**Results:**

The amount of energy needed to match the natural images was consistently (i.e., across all images and subject groups) higher with the contact lenses that contained the HEV‐filter. Additionally, estimated log relative energy differences between HEV‐ and non‐HEV‐filter lenses were 0.13, 0.14 and 0.17 for spherical, multifocal and toric lenses, respectively.

**Conclusion:**

The collection of images appeared ≈12% brighter (averaged across spherical, multifocal and toric lenses on a log scale) when wearing the HEV‐filtering lenses; this translates to about 40% more energy needed to match the comparison field with the adjacent images. These results illustrate that lenses filtering HEV light are capable of increasing the subjective brightness of a naturalistic scene.

**Key points**

- Contact lenses that filter high‐energy visible light can make everyday scenes appear brighter, even though they reduce the overall amount of light reaching the eye.
- By improving brightness perception without glare, high‐energy visible‐filtering contact lenses may support visual performance for a wide range of users, including those with or without eye conditions.
- High‐energy visible light filtering alters perception in ways not explained by light reduction alone, pointing to additional physiological mechanisms with important basic and clinical applications.

## INTRODUCTION

There is a common perception that yellow filters, including high‐energy visible (HEV) filtering lenses, make the world appear brighter. It is common, for instance, in photography, to use coloured filters (e.g., the #12 Tiffen Yellow filter, The Tiffen Company, tiffen.com) to brighten images and sharpen chromatic borders. HEV‐filtering lenses are commonly used by athletes for this purpose (as well as for indications such as reducing glare, extending visual range, etc.)^1^ and in daily life.^2^ HEV‐filtering lenses tend to filter from about 380–455 nm (HEV2 and HEV3)^3^ and some evidence^3^ suggests that they increase the brightness/crispness of outdoor scenes. This general impression is meaningful. Brightness is a subjective variable: if someone thinks or feels that a scene is brighter, then it is brighter by definition. Wolffsohn et al.^4^ tested directly the effects of HEV lenses on brightness perception in 20 young healthy adults. Subjects went outside on a clear day with the sun directly overhead and viewed the local neighbourhood (sky, road, trees, houses, lawns, etc.) through various filters (a clear control and three step filters). They were then asked a series of questions on a 5‐point ordinal scale about brightness and vision quality. The HEV filter (450 nm cut‐off) was the only lens that increased the subject's perception of brightness significantly.

Laboratory studies regarding the effects of HEV filters on brightness have produced more mixed results (reviewed by Luque et al.^5^) and generally use methods and test materials that themselves are somewhat mixed, that is, they test lenses with significantly different spectral filtering profiles. Studies also often use simple stimuli like the bipartite circular stimuli adopted in heterochromatic brightness matching.^6^ Few investigations have tested the effects of HEV filtering on the brightness of complex natural scenes (Wolffsohn et al.^4^ is a notable exception).

Another issue concerns the mechanism*: how does filtering, effectively reducing light input, increase perceived luminance (lightness)/brightness? There are several possibilities. The first is simple chromatic contrast (originally argued by Walls and Judd^7^). Luria^8^ showed that a yellow test flash on a blue background is more visible when viewed through a short‐wave absorbing filter (in addition, see^4^, ^9^). By absorbing one side of a chromatic border more than the other, the contrast between the two is increased. A sharper contrast between an object in the foreground and background could increase brightness. This effect applies well to the type of simple stimuli used in the classic tasks testing heterochromatic brightness matching but is harder to apply to complex scenes with many chromatic borders more or less influenced by HEV filtering. Walls^10^ originally argued, however, that this type of filtering favours most natural scenes:

By cutting out the different amounts of blue in different but alike‐looking green mixtures, the greens are made to look unlike; and almost any other contrasts can be sacrificed by the animal if only those between greens, so numerous in nature, can be enhanced.

It is well known that contrast effects are major drivers of brightness perception.^11^ However, brightness also depends on the saturation of colours (the so‐called Helmholtz‐Kohlrausch effect): the more saturated a colour, the brighter it will appear.^12^ Hence, even spectrally flat filters can potentially increase lightness/brightness of some coloured images.^13^

Kelly^14^ argued a more unitary mechanism: filtering brings rods ‘online’, which boosts the overall chromatic signal (up to 40% in their study). Bringing rods online, effectively, induces a Purkinje shift, shifting peak sensitivity towards the blues/greens, so those colours appear brighter. A final possible mechanism is that HEV filters reduce the activity of cell types (namely short‐wavelength (S) cones and intrinsically photosensitive retinal ganglion cells) that inhibit the luminance channel under certain conditions.^15^, ^16^

In the present study, we hypothesised that HEV‐filtering soft contact lenses would increase the perceived brightness of complex, naturalistic images, even when the overall luminance of the image was held constant. Establishing this effect would clarify the perceptual impact of HEV filtering, address inconsistencies in prior laboratory findings and help explain their widespread use in sports and daily life.

## METHODS

### Study design

A stratified, controlled, prospective, randomised, double‐masked, bilateral, non‐dispensing, crossover study design was used. Participants attended two study visits, separated by a 1–14 day wash‐out period and were randomly assigned to wear a HEV‐filtering contact lens (Test) or a clear lens (Control) on both eyes during the first visit, counterbalanced on the second visit. The overall sample was divided into three subgroups: subjects who habitually wore spherical, multifocal or toric lenses wore the same type of lenses in the study. Subjects were examined by the attending eyecare professional who fitted the lenses according to the control fitting guides for each product. Subjects were then escorted to a different part of the building where the psychophysical testing was performed by masked investigators. This study was conducted at a single site (University of Georgia).

### Subject selection and ethics approval

Adult (*n* = 124, age 18–70 inclusive) subjects of any gender, race and ethnicity who satisfied the eligibility criteria were enrolled. All subjects needed to be habitual wearers of soft contact lenses in both eyes, worn in a daily disposable or daily wear reusable modality for a minimum of 5 days per week and 6 h per day for the preceding 30 days. Habitual wearers of spherical (age 18–39 years), multifocal (age 40–70 years) and toric lens (age 18–39 years) designs must have contact lens powers consistent with those available for the study. Subjects needed to be in good health with no systemic or ocular conditions that might contraindicate or interfere with contact lens wear or study endpoints.

**FIGURE 1:**
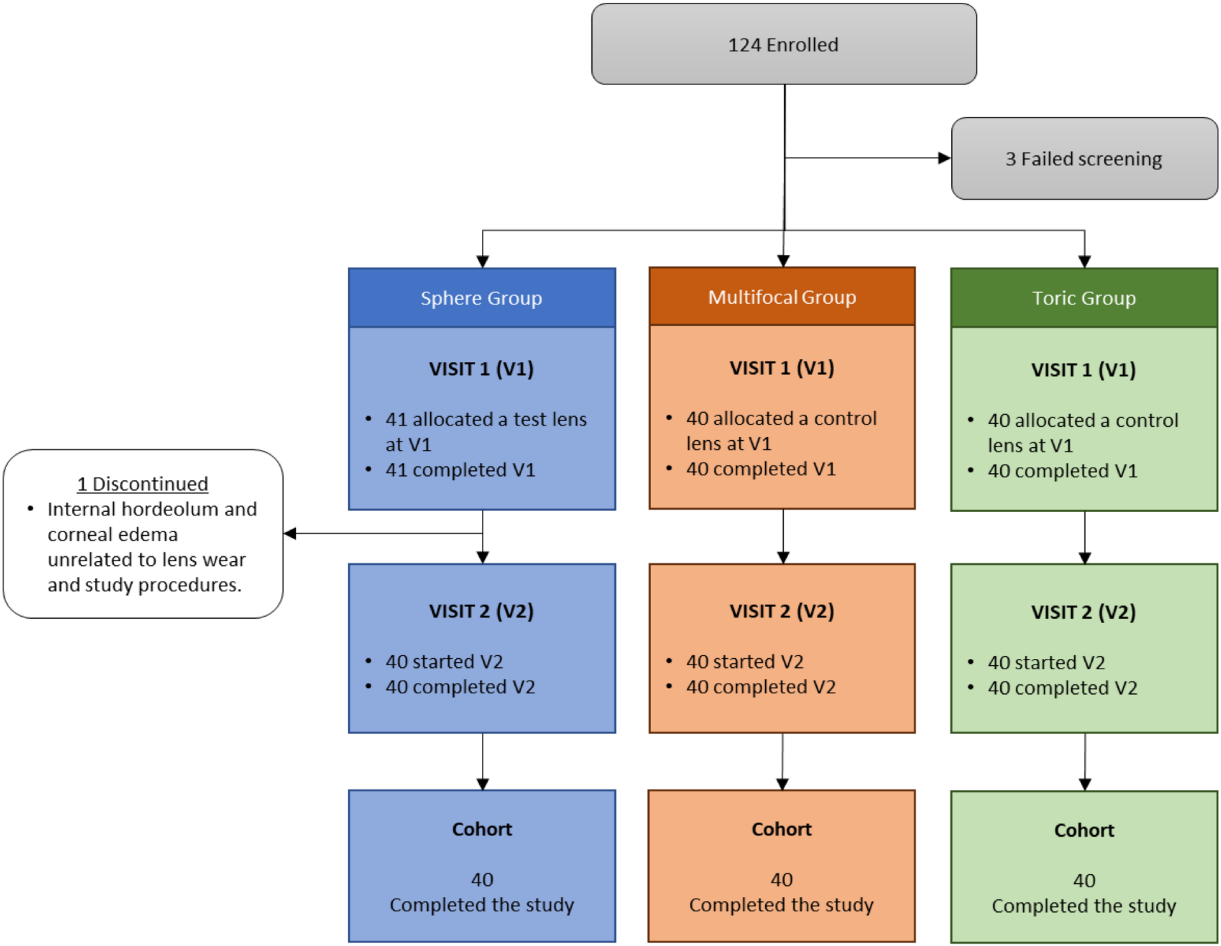
Subject accountability.

The study protocol was approved by an independent Institutional Review Board (Sterling IRB, sterlingirb.com, ID number 10476). This clinical trial was conducted in compliance with the protocol, ISO 14155:2020 Clinical investigation of medical devices for human subjects – Good clinical practice, the Declaration of Helsinki, United States (US) Code of Federal Regulations (CFR) and the International Council for Harmonization Good Clinical Practice E6(R2) (ICH GCP). Participants were informed about the aims and methods of the study, and all signed a statement of informed consent. The study was registered on clinicaltrials.gov (NCT05601544). Figure 1 shows the subject accountability diagram.

### Study lenses

The study lenses consisted of three different designs: spherical, multifocal and toric (Table 1). The control lenses were matched to the test lenses as closely as possible to minimise confounding factors. All lenses were marketed products except for the toric test lens, which was the ACUVUE OASYS MAX 1‐Day for Astigmatism (acuvue.com) prototype. See Figure 1 of Hammond et al.^17^ for transmission spectra of the test and control lenses (or Figure S1). The percentage light filtering of the study lenses is provided in Table 1, consistent with the Accredited Standards Committee (ASC) Z80 Spectral Bands Task Force recommendations.^18^ All transmission data are based on −3.00 DS spherical lens powers, high ADD for multifocal lenses and −0.75 DC × 180 for toric lenses. The transmission spectra of the lenses are shown in Figure S1.

**TABLE 1:** Study lenses.

	Test			Control		
	Spherical	Multifocal	Toric	Spherical	Multifocal	Toric
Name	ACUVUE OASYS MAX 1‐day	ACUVUE OASYS MAX 1‐day multifocal	ACUVUE OASYS MAX 1‐day for astigmatism	ACUVUE OASYS 1‐day	ACUVUE OASYS multifocal	ACUVUE OASYS 1‐day for astigmatism
Material	Senofilcon A	Senofilcon A	Senofilcon A	Senofilcon A	Senofilcon A	Senofilcon A
Base curve (mm)	8.5	8.3	8.5	8.5	8.4	8.5
Diameter (mm)	14.3	14.3	14.3	14.3	14.3	14.3
Sphere power (D)	−1.00 through −6.00 in 0.25 steps	−1.00 through −6.00 in 0.25 steps	−1.50 through −4.00 in 0.25 steps	−1.00 through −6.00 in 0.25 steps	−1.00 through −6.00 in 0.25 steps	−1.50 through −4.00 in 0.25 steps
Cylinder power (D)	NA	NA	−0.75, −1.25	NA	NA	−0.75, −1.25
Cylinder axes (degrees)	NA	NA	10, 80, 90, 100, 170, 180	NA	NA	10, 80, 90, 100, 170, 180
ADD power (D)	NA	Low, Med, High	NA	NA	Low, Med, High	NA
Visible spectrum filtering (%). 380–780 nm	13	13	14	3	3	3
HEV‐1 filtering (%). 455–500 nm	2	2	3	1	1	1
HEV‐2 filtering (%). 400–455 nm	47	46	49	2	2	3
HEV‐3 filtering (%). 380–400 nm	100	99	100	25	22	28
Blue–violet filtering (%). 380–450 nm	65	64	67	9	8	10

*Note*: All were manufactured by Johnson and Johnson (acuvue.com).

Abbreviations: HEV, high‐energy visible wavelengths; NA, not applicable.

### Apparatus and procedure

**FIGURE 2:**
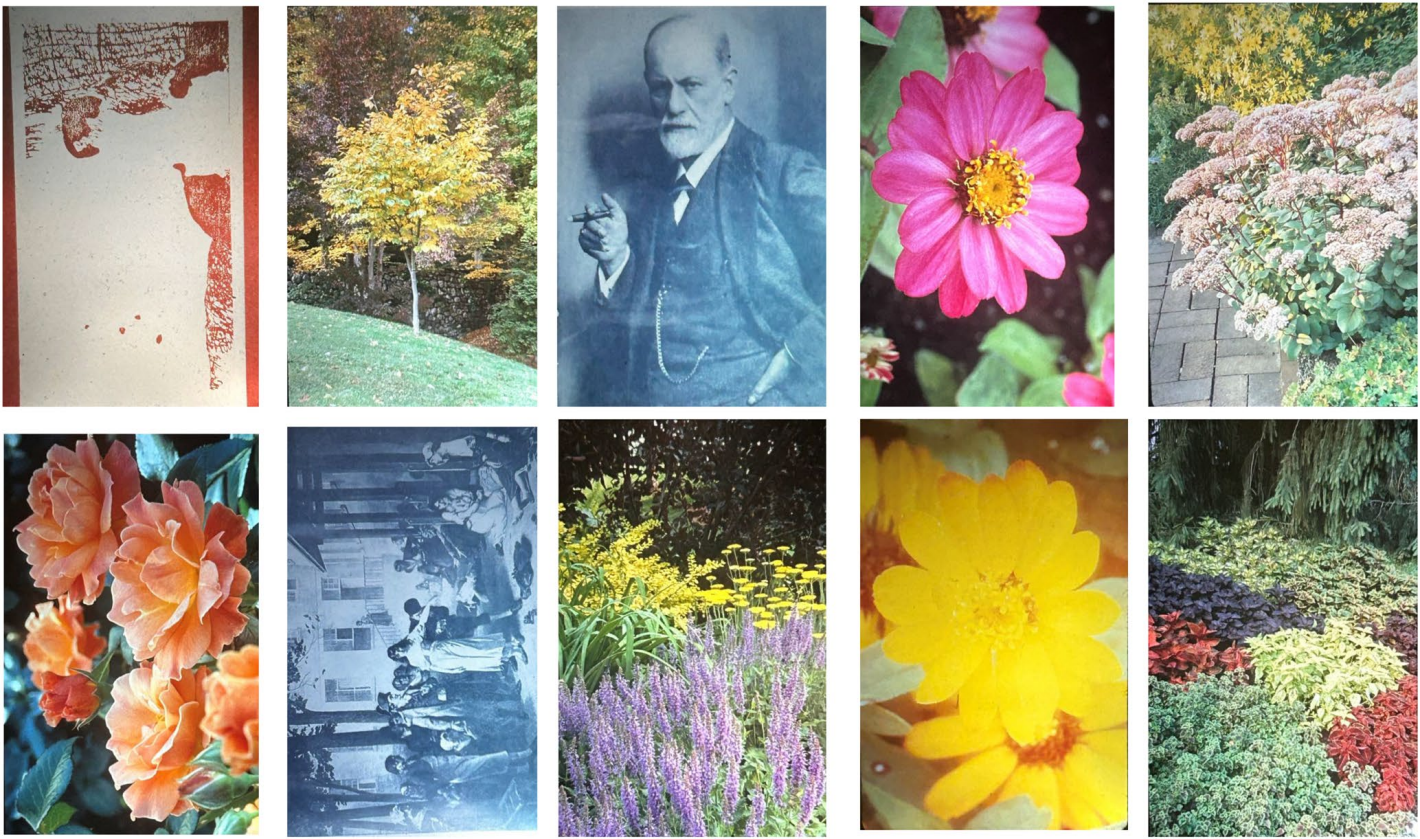
The 10 test images that were used.

Brightness perception was measured based on the classic principle of heterochromatic brightness matching. However, rather than a simple bipartite field, subjects viewed a yellow comparison field to match the brightness of a variety of natural images. The 10 images that were used are shown in Figure 2.

**FIGURE 3:**
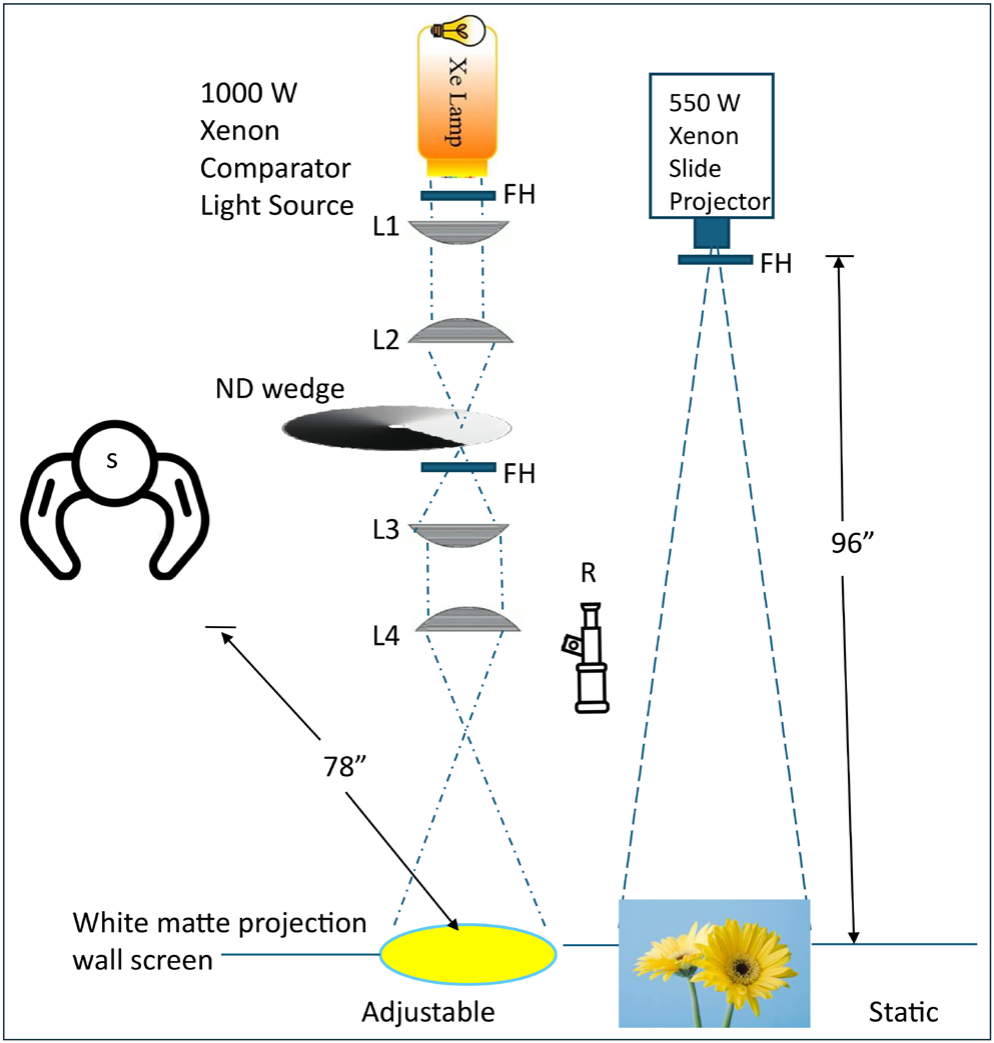
Experimental setup (FH, filter holders; L, achromatic plano‐convex coated lenses; ND, neutral density; R, radiometer; S, subject).

The comparison field was broadband but short‐wave deficient (created by a yellow Corning 51,300 glass filter; Corning Specialty Glass, corning.com). The spectrum of the comparison field (shown in Figure S1) was chosen to be unaffected by the test lens (neither the control nor test lens filtered the wavelengths in the comparison field). If this image (fixed intensity) appeared brighter to the subject (for either the test or control lens), then the intensity of the comparison field was adjusted to match the image (as part of the procedure, the comparison field was made both dimmer and brighter to achieve the best match). The images (ranging from chromatic natural scenes to achromatic patterns) were roughly rectangular (~15°) and were created by a xenon‐based (550‐Watt) slide projector (Navitar Xenon 560; Navitar Inc.; solarisnetwork.com), which projected the images onto a matte‐white wall screen. A neutral density filter placed in front of the projection lens was used to adjust the absolute intensity (averaged around 16 cd/m^2^ at the screen) of the image (so there was ample room for the comparison field to be significantly dimmer and brighter). The intensity was checked in each experimental session with a mounted luminance spot meter (Gossen Mavo‐Spot 2 USB; Gossen Metrowatt USA; gossenmetrawattusa.com) to confirm stability throughout the study (also used to confirm the intensity of the comparison field when at a set intensity). The circular 15‐degree comparison field was created with a single channel parallel optical system. This system used a 1000‐watt xenon arc (Newport Corporation; newport.com/f/research‐arc‐lamp‐sources) as the light source, achromatic lenses (the latter being a focusing lens that projected the image onto the wall screen), the yellow Corning filter (to set the chromatic content of the comparison field) and a circular neutral density wedge to adjust the intensity (Figure 3).

The subjects, seated 78 inches (approximately 198.1 cm) away from the screen, indicated when the comparison matched the brightness of the image (Method of Limits). First, subjects were shown the comparison when it looked much dimmer and much brighter than the natural image. Then the comparison field was slowly increased or decreased by the investigator until the subject indicated the brightness matched. The intensity of the comparison field was recorded as the amount of light transmitted and expressed as log relative energy. Three ascending/descending trials were collected for each image.

### Statistical methods

Statistical analyses were conducted using the Statistical Analysis System (SAS) software, Version 9.4 (SAS Institute; sas.com). The brightness perception score was calculated as the average log relative energy (LRE) for the top five scenes for each subject, where the top five scenes were defined as the images with the highest log relative energy for each subject. This score was analysed separately for three subject groups (spherical, multifocal and toric wearers) using a linear mixed model. The decision to concentrate on the top five scenes was established before the unmasked data was viewed/available, ensuring that a priori predictions were not influenced by any results or significance statements that may have emerged later. The differences observed between the test and control groups, however, remained consistent when comparing both adjusted (top five scenes) and unadjusted (all scenes) brightness perceptions.

Each model included lens type (categorised as test or control), lens wear sequence, period, age, gender, race (categorised as white or non‐white) and iris category (categorised as dark or light) as fixed effects. An unstructured covariance matrix was employed to model the residual errors for within‐subject repeated measures. The significance level for the brightness perception analyses was determined a priori in the clinical study protocol to appropriately control Type I error. For spherical and multifocal wearers, brightness perception was included among the secondary/post hoc hypotheses. Since these were evaluated alongside other secondary endpoints, the alpha was adjusted using a Bonferroni correction (1‐sided 0.025 ÷ 3 = 0.0083), yielding a 2‐sided threshold of approximately 0.0167. For toric wearers, brightness perception was designated as an exploratory endpoint. Accordingly, it was analysed without multiplicity adjustment, using the conventional 0.05 significance level. Secondarily, a linear mixed model was applied to the log‐transformed brightness perception score (on the original LRE scale) to estimate the percentage improvement in LRE between the HEV‐filter and non‐HEV‐filter lenses for each subject group. The fixed effects, covariance structure and significance thresholds used in the original LRE scale analysis were maintained for the log‐transformed models.

Lastly, the brightness perception score was back transformed to the linear scale, and a linear mixed model was applied to the log‐transformed values to derive the percentage of improvement on the linear scale for each subject group. The fixed effects, covariance structure and significance thresholds used in the original LRE scale analysis were maintained for the log‐transformed linear scale models.

## RESULTS

### Subject accountability and demographics

Among the 124 subjects enrolled, 121 (97.6%) subjects were randomly assigned to one of two unique lens wear sequences (Test/Control or Control/Test), were fitted with at least one study lens and were included in the safety population and intent‐to‐treat (ITT) population, while three (2.4%) subjects failed to meet all eligibility criteria. Of these 121 subjects in the ITT population, 41 (33.9%) were fitted with spherical lenses, 40 (33.1%) were fitted with multifocal lenses, and 40 (33.1%) were fitted with toric lenses. One subject was discontinued between the first and second visits (sphere group) due to a hordeolum and corneal oedema in their right eye, unrelated to study lenses or procedures (See the Consort Diagram in Figure 1).

Overall, of the 121 subjects from the ITT population, 80% were female, 82% were white, and 94% were not Hispanic or Latino. Their average age was 33.8 (±13.6) years. All 121 subjects were from the USA. A breakdown of subject demographics by study group is shown in Table 2.

**TABLE 2:** Subject demographics for each lens type.

Characteristic	Spherical (*N* = 41)	Multifocal (*N* = 40)	Toric (*N* = 40)	All (*N* = 121)
Gender *n* (%)	Female: 29 (70.7)	Female: 37 (92.5)	Female: 30 (75.0)	Female: 96 (79.3)
	Male: 12 (29.3)	Male: 3 (7.5)	Male: 10 (25.0)	Male: 25 (20.7)
Race *n* (%)	Asian: 6 (14.6)		Asian: 2 (5.0)	Asian: 8 (6.6)
	Black: 2 (4.9)	Black: 5 (12.5)	Black: 4 (10.0)	Black: 11 (9.1)
	White: 32 (78.0)	White: 34 (85.0)	White: 33 (82.5)	White: 99 (81.8)
	Multiple: 1 (2.4)	Multiple: 1 (2.5)	Multiple: 1 (2.5)	Multiple: 3 (2.5)
Ethnicity *n* (%)	Hispanic: 5 (12.2)	Hispanic: 0 (0.0)	Hispanic: 2 (5.0)	Hispanic: 7 (5.8)
	Not: 36 (87.8)	Not: 40 (100)	Not: 38 (95.0)	Not: 114 (94.2)
Age (years)	Mean (SD): 25.8 (6.5)	Mean (SD): 51.0 (7.1)	Mean (SD): 24.9 (5.3)	Mean (SD): 33.8 (13.6)
	Median: 23	Median: 48.5	Median: 24	Median: 30
	Range: 18–39	Range: 40–68	Range: 18–39	Range: 18–68
Iris colour *n* (%)	Dark: 25 (61.0)	Dark: 19 (47.5)	Dark: 26 (65.0)	Dark: 70 (57.9)
	Light: 16 (39.0)	Light: 21 (52.5)	Light: 14 (35.0)	Light: 51 (42.1)
ADD group *n* (%)	Not applicable: 41 (100)	Low: 10 (25.0)	Not applicable: 40 (100)	Low: 10 (25.0)
		Mid: 12 (30.0)		Mid: 12 (30.0)
		High: 18 (45.0)		High: 18 (45.0)

### Brightness matching

**FIGURE 4:**
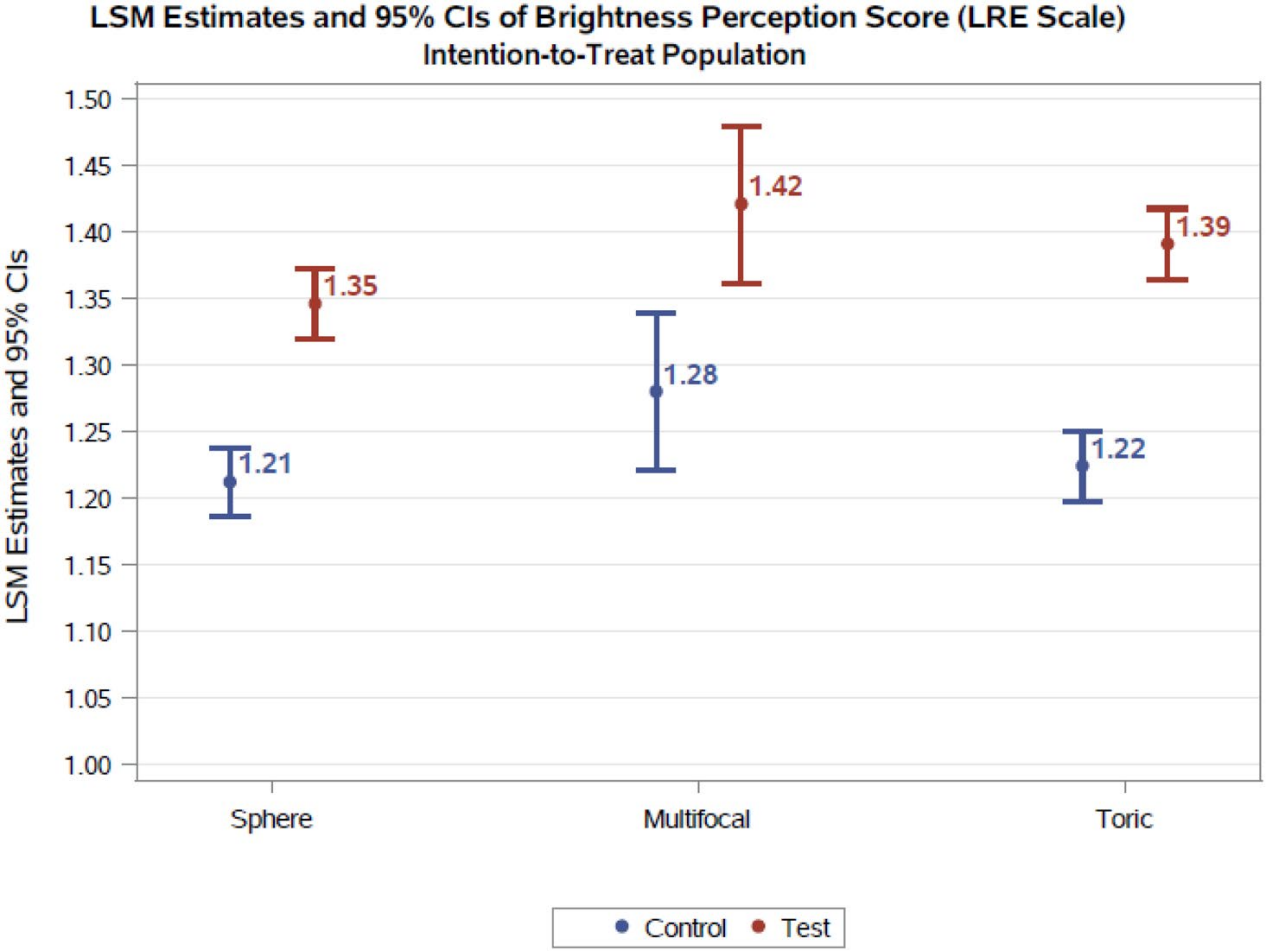
Least‐squares mean (LSM) estimates of brightness perception (expressed as log relative energy). CI, confidence interval; LRE, log relative energy.

Table 3 lists the observed log relative energy needed in the comparison field to match each image that was presented. Figure 4 shows the estimated brightness perception score (on LRE scale) by subject group and study lens. The magnitude of this effect was numerically different across the three groups, but the difference was not statistically significant (*p* = 0.40 for comparisons across the spherical, multifocal and toric test lenses; *p* = 0.69 for comparisons across the spherical, multifocal and toric control lenses).

**TABLE 3:** Brightness perception (log relative energy).

Image	Spherical C	Spherical T	Multifocal C	Multifocal T	Toric C	Toric T
*n*	41	40	40	40	40	40
Orange flower	1.212 (0.08)	1.334 (0.09)	1.217 (0.11)	1.359 (0.09)	1.224 (0.10)	1.358 (0.06)
Bushes	1.159 (0.10)	1.306 (0.08)	1.186 (0.12)	1.313 (0.13)	1.152 (0.11)	1.327 (0.07)
Fall tree	1.139 (0.09)	1.316 (0.09)	1.148 (0.12)	1.288 (0.15)	1.164 (0.07)	1.325 (0.08)
Yellow and purple flowers	1.137 (0.09)	1.313 (0.08)	1.131 (0.15)	1.294 (0.16)	1.129 (0.11)	1.325 (0.08)
Pink and yellow flowers	1.218 (0.08)	1.354 (0.07)	1.192 (0.11)	1.338 (0.11)	1.198 (0.10)	1.373 (0.08)
Purple with Yellow flower	1.158 (0.10)	1.303 (0.09)	1.183 (0.11)	1.324 (0.12)	1.169 (0.11)	1.342 (0.06)
Orange‐cream flowers	1.152 (0.09)	1.316 (0.09)	1.142 (0.16)	1.313 (0.12)	1.144 (0.11)	1.345 (0.06)
Town square	1.163 (0.12)	1.298 (0.11)	1.105 (0.13)	1.262 (0.10)	1.129 (0.13)	1.337 (0.08)
Abstract image	1.148 (0.10)	1.284 (0.11)	1.094 (0.14)	1.222 (0.15)	1.149 (0.10)	1.322 (0.10)
Sigmund Freud	1.145 (0.11)	1.259 (0.10)	1.052 (0.15)	1.204 (0.13)	1.106 (0.13)	1.304 (0.10)
Average	1.164 (0.07)	1.309 (0.07)	1.145 (0.09)	1.292 (0.10)	1.157 (0.08)	1.336 (0.05)
Top‐5 adjusted average	1.222 (0.06)	1.356 (0.06)	1.219 (0.08)	1.360 (0.08)	1.216 (0.06)	1.383 (0.04)

*Note*: All values are Mean (SD) in log relative energy units. *n* values identical across all images: Spherical (41 C/40 T), Multifocal (40 C/T), Toric (40 C/T).

Abbreviations: C, control lens; T, test lens.

As shown in the tables and Figure 4, when participants wore the HEV‐filtering lenses (compared to the same subjects wearing the clear lenses in randomised order), they needed about 40% more energy in the comparison field to match the brightness in the test image. This translates to about a 12% difference in brightness (log scale).

## DISCUSSION

This study was double‐masked and bilateral (i.e., the test or control lens was placed on both eyes by the attending clinician while the experimenter and subjects remained naïve as to the intervention). Subjects were asked to indicate when a homogeneous yellow field was equally bright to a set of images; brightness matching was performed using both eyes. This homogeneous field could be adjusted to be much dimmer or brighter than the test image. Despite the subjective nature of the task (indicating when the two fields were equally bright), the subjects' performance was surprisingly consistent; both across lens type (spherical, toric and multifocal) and in favour of the lens containing the HEV filter. As shown in Table 3 and Figure 4, the estimated log brightness perception score difference (test minus control) averaged across the subject group (spherical, multifocal and toric wearers) was about 0.15. When wearing the HEV‐filtering lenses, subjects needed about 40% more energy to match the two fields (Table 4).

**TABLE 4:** Percentage brightness improvement across lens type.

Percentage brightness score improvement (test‐control)					
Log relative energy scale			Intensity of the comparison field (linear scale) needed for the match		
Spherical	Multifocal	Toric	Spherical	Multifocal	Toric
11.0%	11.6%	13.8%	36.1%	38.3%	46.9%

These results are consistent with the common impression that HEV filters are capable of increasing the subjective brightness of a naturalistic scene. Studies using magnitude estimation have shown that brightness is non‐linear. On the lower luminance side, small changes in stimulus intensity can result in relatively large changes in brightness perception. However, when the stimulus is sufficiently intense, changes in perceived brightness quickly plateau (this is why filtering of any kind would not make an image uncomfortably bright).

In this study, the improvement in brightness found when wearing HEV‐filtering lenses was similar across images (even those with no chromatic borders). The uniformity of the effect suggests that the mechanism underlying the effect was likely more physiological than optical per se. One possibility is that HEV‐filtering lenses boost the luminance signal by bringing rods online (as suggested by Kelly^14^). If so, it may be due to the fact that HEV filters mimic spectral changes that occur naturally throughout the day. The correlated colour temperature (CCT) during the middle of the day (~6500 K) contains more short‐wave energy than near dusk (~3000 K). In other words, wearing a HEV filter is analogous to simulating the conditions that induce a Purkinje shift. Other physiological changes are also possible. Short‐wave filtering can decrease the activity of S‐cones^15^ and melanopsin,^16^ which may have reduced inhibitory effects on the luminance channel under the conditions of the present experiment. It is important to note, however, that a limitation of this study is that it did not test the mechanisms underlying the effects observed (i.e., interpretations are speculative). Nonetheless, this study, like others,^19^ suggests that higher‐level percepts (like brightness) can be altered significantly based on lower‐level input. This conclusion, however, must also be tentative. Higher‐level percepts do not tend to be isolated. This study used images in a controlled laboratory setting projected with a xenon slide projector. In a real‐world environment, scenes are dynamic with varying lighting, movement and depth cues (see Table A1 in Appendix A).

In previous studies,^20^, ^21^ we have shown that this type of HEV‐filtering contact lens reduces glare discomfort, disability and positive dysphotopsias. The present study showed that HEV filters increase subjective brightness. Individuals with conditions such as early cataracts, age‐related macular degeneration (AMD) or photophobia might benefit from HEV‐filtering lenses that brighten scenes without increasing glare issues.

## CONCLUSION

This double‐masked, bilateral study demonstrated that HEV‐filtering lenses consistently increased subjective brightness perception across different lens types, with wearers requiring ~40% more comparison field energy to achieve brightness matches (analogous to the magnitude of effect seen by Kelly^14^). The effect was uniform across images, suggesting a physiological rather than purely optical mechanism, potentially involving rod recruitment or modulation of short‐wavelength pathways. Although the exact mechanism remains speculative, these findings support the common impression that HEV filters enhance perceived brightness without inducing glare. Clinically, such lenses could potentially provide functional benefits for individuals with early cataracts, AMD or photophobia by brightening scenes in a comfortable manner.

## AUTHOR CONTRIBUTIONS

**Billy R. Hammond:** Conceptualization (lead); data curation (equal); formal analysis (supporting); funding acquisition (equal); investigation (lead); methodology (lead); project administration (equal); resources (equal); supervision (equal); validation (equal); visualization (equal); writing – original draft (lead); writing – review and editing (equal). **John R. Buch:** Conceptualization (equal); funding acquisition (equal); investigation (equal); resources (equal); visualization (equal); writing – review and editing (equal). **Patricia Martin:** Conceptualization (equal); resources (equal); writing – review and editing (equal). **Wright Shamp:** Data curation (supporting); formal analysis (lead); software (equal). **Jacob B. Harth:** Investigation (equal); methodology (equal); project administration (equal); supervision (equal); writing – review and editing (equal). **Cameron Wysocky:** Data curation (supporting); investigation (supporting); project administration (supporting). **Lisa M. Renzi‐Hammond:** Conceptualization (equal); data curation (equal); formal analysis (supporting); funding acquisition (equal); investigation (equal); methodology (supporting); project administration (lead); resources (equal); validation (equal); writing – original draft (supporting); writing – review and editing (equal).

## FUNDING INFORMATION

This study was supported by Johnson & Johnson Vision Care Inc.

## CONFLICT OF INTEREST STATEMENT

Lisa M. Renzi‐Hammond and Billy R. Hammond have worked as consultants for Johnson & Johnson Vision Care, the primary funder of this research. John Buch, Patricia Martin and Wright Shamp are employed full‐time by Johnson and Johnson Vision Care.

## PATIENT CONSENT STATEMENT

Written informed consent was obtained from all participants.

## CLINICAL TRIAL REGISTRATION

clinicaltrials.gov (NCT05601544).

## Supporting information


Figure S1.

## Data Availability

Individual data are available upon reasonable request.

## Footnotes

* The mechanism is not just dependent on spectral filtering but also site. For example, contact lenses differ from spectacles in that they move with the eye and are worn for longer periods. Hence, subjects would adapt to filtering by a contact lens (or an intraocular implant) in a way that they would not to spectacles that are more often removed. Contact lenses would differ from the macular pigments in that contacts could screen the entire retina whereas the macular pigments would mostly only screen cones within the macula.

## APPENDIX A

**TABLE A1:** LSM estimates and 95% CIs of Brightness Perception Score (average log relative energy [LRE] Scale).

Lens design	Lens	LSM	Standard error	Two‐sided 95% confidence interval
Spherical	Control	1.21	0.013	(1.19, 1.24)
	Test	1.35	0.013	(1.32, 1.37)
Multifocal	Control	1.28	0.029	(1.22, 1.34)
	Test	1.42	0.029	(1.36, 1.48)
Toric	Control	1.22	0.013	(1.20, 1.25)
	Test	1.39	0.013	(1.36, 1.42)

Abbreviations: CI, confidence interval; LSM, least‐squares mean.

## References

[1] Harth JB , Renzi‐Hammond LM , Hammond BR Jr . A dietary strategy for optimizing the visual range of athletes. Exerc Sport Sci Rev. 2023;51:103–108. 37083620 10.1249/JES.0000000000000318 PMC10259207

[2] Aoki K , Kohmura Y , Murakami S , Someya Y . Effects of yellow‐tinted lenses on visual attributes related to sports activities and daily life in late middle‐aged adults. Cent Eur J Sport Sci Med. 2015;9:27–36. 10.2478/hukin-2013-0003 PMC3661891 23717352

[3] American National Standards Institute . ASC Z80 SBTF Technical Report. 2023. https://thevisioncouncil.org/sites/default/files/assets/media/ASC%20Z80%20SBTF%20Technical%20Report%20FINAL%2029NOV2023.pdf. Accessed 27 Oct, 2025.

[4] Wolffsohn JS , Cochrane AL , Khoo H , Yoshimitsu Y , Wu S . Contrast is enhanced by yellow lenses because of selective reduction of short‐wavelength light. Optom Vis Sci. 2000;77:73–81. 10701805 10.1097/00006324-200002000-00011

[5] Luque MJ , Capilla P , Diez MA , Felipe A . Effect of a yellow filter on brightness evaluated by asymmetric matching: measurements and predictions. J Opt A Pure Appl Opt. 2006;8:398–408.

[6] Long F , Yang Z , Purves D . Spectral statistics in natural scenes predict hue, saturation and brightness. Proc Natl Acad Sci U S A. 2006;103:6013–6018. 16595630 10.1073/pnas.0600890103 PMC1426241

[7] Walls GL , Judd HD . The intra‐ocular colour‐filters of vertebrates. Br J Ophthalmol. 1933;17:641–675. 18169162 10.1136/bjo.17.11.641 PMC511614

[8] Luria SM . Vision with chromatic filters. Optom Vis Sci. 1972;49:818–829. 10.1097/00006324-197210000-00002 4564949

[9] Renzi LM , Hammond BR . The effect of macular pigment on heterochromatic luminance contrast. Exp Eye Res. 2010;91:896–900. 20883691 10.1016/j.exer.2010.09.015

[10] Walls GL . The vertebrate eye and its adaptive radiation. New York, New York: Hafner Publishing; 1942.

[11] Zaidi Q , Yoshimi B , Flanigan N , Canova A . Lateral interactions within color mechanism in simultaneous induced contrast. Vision Res. 1992;32:1695–1707. 1455741 10.1016/0042-6989(92)90162-c

[12] Corney D , Haynes JD , Rees G , Lotto RB . The brightness of colour. PLoS One. 2009;4:e5091. 10.1371/journal.pone.0005091 19333398 PMC2659800

[13] de Ridder H . Naturalness and image quality: saturation and lightness variation in color images of natural scenes. J Imaging Sci Technol. 1996;40:487–493.

[14] Kelly SA . Effect of yellow‐tinted lenses on brightness. J Opt Soc Am A. 1990;7:1905–1911. 2231102 10.1364/josaa.7.001905

[15] Wade AR . Long‐range suppressive interactions between S‐cone and luminance channels. Vision Res. 2009;49:1554–1562. 19344735 10.1016/j.visres.2009.03.023 PMC2703610

[16] Yamakawa M , Tsujimura SI , Okajima K . A quantitative analysis of the contribution of melanopsin to brightness perception. Sci Rep. 2019;9:7568. 10.1038/s41598-019-44035-3 31110303 PMC6527610

[17] Hammond BR , Buch J , Renzi‐Hammond LM , Bosten JM , Nankivil D . The effect of a short‐wave filtering contact lens on color appearance. J Vis. 2023;23:2. 10.1167/jov.23.1.2 PMC9819670 36595282

[18] ASC Z80 Spectral Bands Task Force . Spectral Bands Task Force Technical Report. 2023. https://thevisioncouncil.org/sites/default/files/assets/media/Spectral%20Band%20Task%20Force%20Technical%20Report%20Press%20Release%2012‐08‐2023%20‐%20FINAL.pdf. Accessed 27 Oct, 2025.

[19] Shapiro A , Lu ZL . Relative brightness in natural images can be accounted for by removing blurry content. Psychol Sci. 2011;22:1452–1459. 22020976 10.1177/0956797611417453

[20] Renzi‐Hammond LM , Buch J , Xu J , Hammond BR . The influence of HEV‐filtering contact lenses on behavioral indices of glare. Eye Contact Lens. 2022;48:509–515. 36201639 10.1097/ICL.0000000000000944 PMC9668403

[21] Renzi‐Hammond LM , Buch J , Xu J , Hammond BR . Reduction of glare discomfort and photostress recovery time through the use of a high‐energy visible–filtering contact lens. Eye Contact Lens. 2022;48:516–520. 36083159 10.1097/ICL.0000000000000935 PMC9668378

