# Supplementary material for: The effects of a HEV‐filtering contact lens on the brightness of natural images
Source: Ophthalmic Physiol Opt. 2025 Oct 29;45(7):1801–10. doi: 10.1111/opo.70035 (PMC12682107; doi:10.1111/opo.70035)
Supplement: Supplementary file 1 — Figure S1. [file OPO-45-1801-s001.pdf]

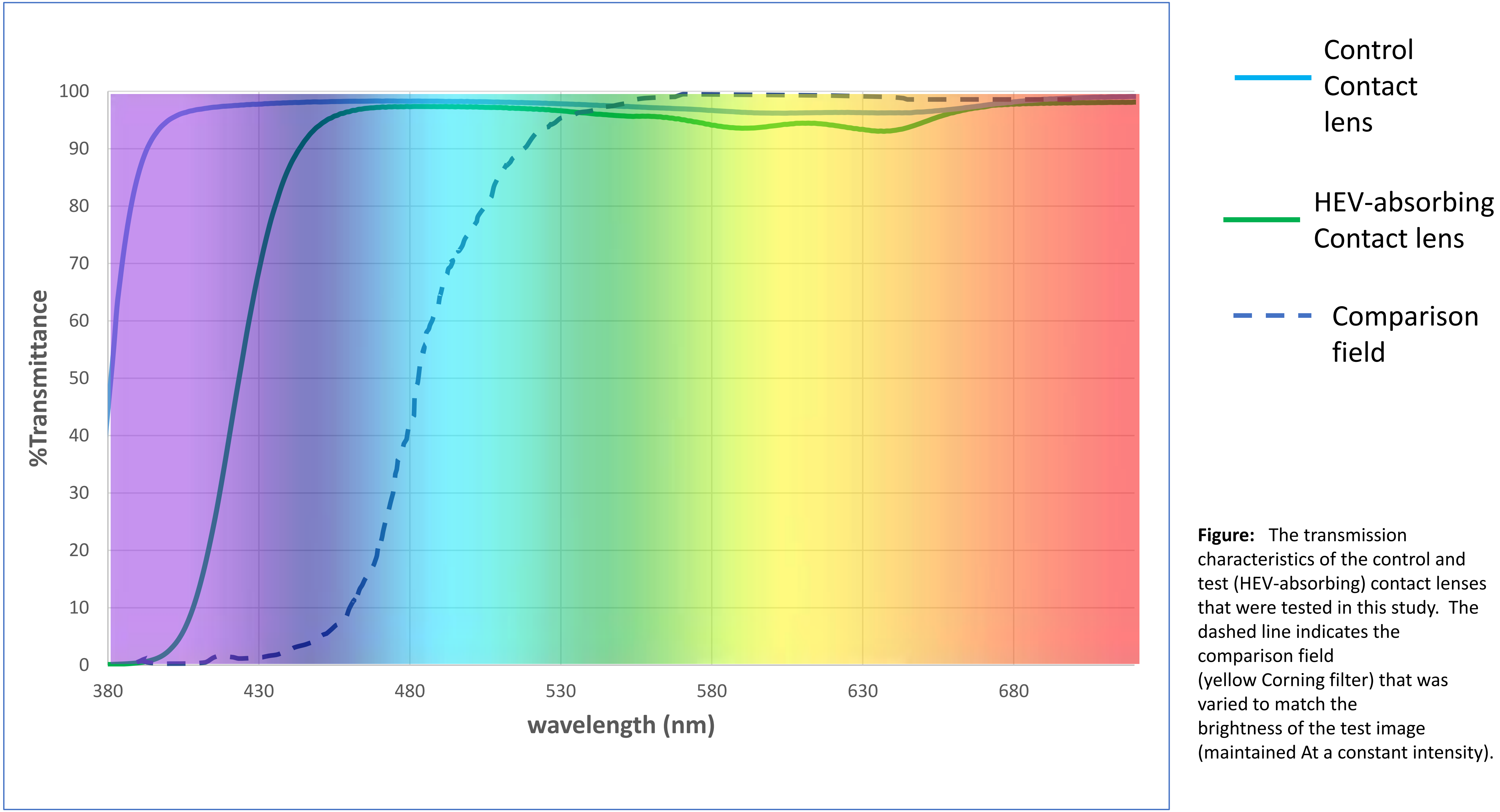

Figure: The transmission characteristics of the control and test (HEV-absorbing) contact lenses that were tested in this study. The dashed line indicates the comparison field (yellow Corning filter) that was varied to match the brightness of the test image (maintained At a constant intensity).
